# Q&A: Life, synthetic biology and risk

**DOI:** 10.1186/1741-7007-8-77

**Published:** 2010-06-14

**Authors:** Steven A Benner

**Affiliations:** 1 Foundation for Applied Molecular Evolution, PO Box 13174, Gainesville, FL 32604, USA

## Recent achievements in synthetic biology have raised the question of what we mean by 'life'. Is a definition possible?

Yes, one can always write out a definition for an abstract concept like 'life'. But a definition has value only if it is set within the context of a theory that gives its terms meaning, as Carol Cleland and Chris Chyba argue in their paper published in 2002. And a definition is most useful if it provides what a scientist needs.

## For example?

Well, water can be defined as a molecule built from two atoms of hydrogen and one atom of water. But this definition must be set in the context of atomic theory from chemistry to have meaning. Further, for a scientist wishing to identify water, this definition may be less useful than an operational definition - for example, that water is a substance that freezes at 0°C, boils at 100°C, has a density of 1 gram per cubic centimeter, and the like.

## How does this apply to life?

Consider this definition from a group of scientists empanelled by NASA in 1994 who suggested that life could be defined as a 'self-sustaining chemical system capable of Darwinian evolution'.

## Self-sustaining? But doesn't most life need to eat something from outside itself?

Yes. But the panelists, when asked, pointed out that 'self-sustaining' was not used to mean that the life must not eat. Rather, the term means only that life must not need to be provided its sustenance through the action of an intelligent being, a gardener or a keeper.

## Aren't you just defining life as we know it? Is this not a bit Earth-o-centric?

This definition is grounded in a deeply held theory of life, as I argue in my book *Life, the Universe and the Scientific Method*. It tells us what we definers believe about what is possible in reality and what is not. Thus, we can conceive, and many science fiction authors have conceived, of life made from pure energy or not requiring Darwinian evolution to exist. Surely, if we encountered such beings during a real (not fictional) star trek, and if they were to talk to us (as aliens generally do in science fiction), we would instantly revise our definition to include them. We do not do so now because we do not believe that they could possibly exist. That is, we believe that anything that has the attributes of life would be chemical and would have come to exist via Darwinian evolution. Admittedly, those beliefs are based on our knowledge of Earth life, and of no other.

## Are those beliefs justifiable from any other perspective?

One can do an interesting thought experiment. In a few years, we may be able to identify DNA sequences that prospectively help our children survive, and gain the technology that allows these sequences to be placed into our germ lines to generate mutant children that are fitter by design. If this happens, then our species will start to escape Darwinian mechanisms for improving our genes. The good news is that we will no longer need to see children die of genetic disease; a large number of bad mutations is the Darwinian cost of a few good ones. With gene therapy, we may imaginably be able to scan the germ line for deleterious genes and remove them.

## Will this mean that we are no longer 'life'?

By this definition, yes: Darwinian evolution does not allow prospective mutation. Through this technology, humankind would be able to evolve in a more Lamarckian way. But this scenario is not implausible. So perhaps we should start thinking now about a better definition-theory of life.

## Well, I see the problem. But is the NASA definition useful?

Cleland and Chyba argue that it is not, taking it to imply that explorers, let us say on Mars, would need to observe a possible life form for years, waiting for it to evolve before they could be certain that it is life. I disagree, since we can define chemical structures necessary to support Darwinian evolution from first principles, and look for those structures. For example, within the 'second-generation' theory of the gene, my group and I argued that a universal genetic molecule in water must have a repeating charge in its backbone, as this is the only kind of molecule that can robustly support Darwinian evolution.

## What is this 'second-generation' theory of the gene?

The 'first-generation theory' was proposed by Watson and Crick in 1953, and is now taught in every high school. Here, DNA is modeled as a ladder having rungs of uniform size because big purines pair with small pyrimidines, hydrogen bonds hold the pairs together, and the sugar-phosphate backbone is largely incidental to strand-strand binding, providing simple scaffolding. Efforts by synthetic biologists to make alternative kinds of DNA have led us to discover that the sugars and the phosphates are integral to the molecular recognition phenomenon. For example, the repeating backbone charge drives strand-strand interactions away from the backbone and towards useful places, keeping single strands largely extended in the process.

## And the repeating charges are useful for Darwinian evolution ... how?

To support Darwinian evolution, a genetic molecule must be able to change the details of its structure without changing its overall biophysical behavior. This ability to change structure but not behavior is actually very rare in molecular systems. Take proteins, for example. A single amino acid replacement can cause a previously soluble protein to precipitate, as in sickle cell anemia. This would be fatal in a genetic molecule. However, a repeating backbone charge dominates the biophysics of a DNA polymer so much that one can change its sequence without changing its biophysical properties. And this allows the biopolymer to support evolution.

## Does the bacterium that Craig Venter made help us to define life?

Not really. Craig Venter's bug is essentially the same as a bacterium that came to us through Darwinian evolution, which provided all of its genetic information. Venter's bug is alive and is life, but it is not particularly new in either of these features. Its DNA is fully synthetic, but the information within its sequence is natural. Likewise, the casing - the cell in which it replicates and instructs protein synthesis - was taken preassembled from an existing cell. Nevertheless, this synthesis fits nicely into the century-long tradition of natural products synthetic chemistry. Natural products chemists first analyze the structure of a biomolecule to determine the arrangement of its constituent atoms, and then synthesize exactly the same biomolecule from scratch. This was first a way to confirm the structural assignment. Later, making bigger and bigger molecules was a way to set 'grand challenges' to test chemistry and its theories. Indeed, any field that allows synthesis of new forms of its subject matter allows ideas and hypotheses to be much more directly tested than in fields limited to observation and analysis.

## Aren't there serious dangers in making synthetic forms of life?

**Figure 1:**
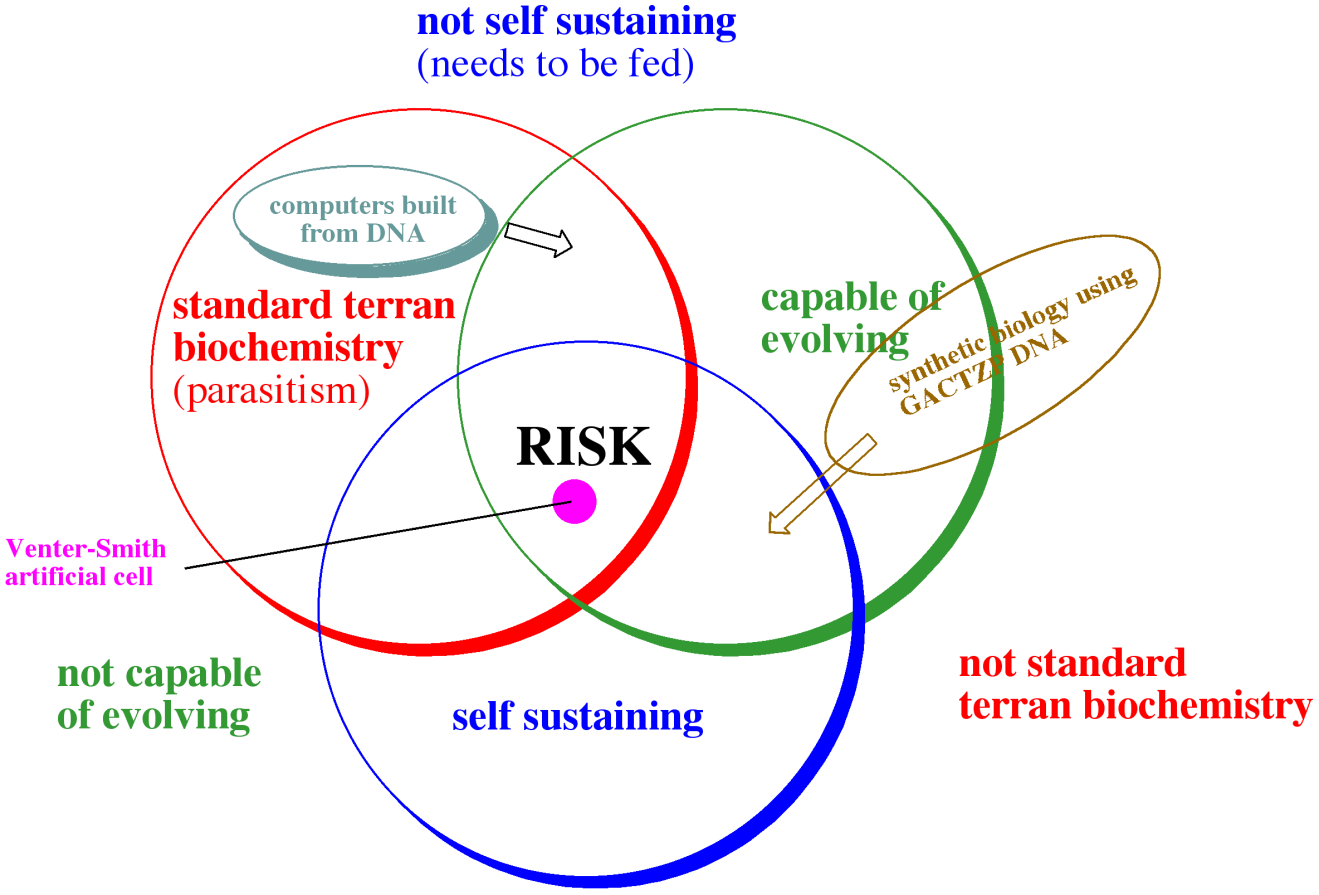
**Potential for danger from synthetic life**. Synthetic life forms display different levels of risk, according to whether they use standard terran biochemistry (inside the red circle), and/or are capable of evolving (inside the green circle) and/or are self-sustaining (inside the blue circle). The arrows indicate the direction that current work is taking the indicated example of synthetic biology. Adapted from Benner SA: *The Life, the Universe and the Scientific Method*. Gainesville: FfAME Press; 2009.

I published a Venn diagram in *Life, the Universe, and the Scientific Method *to illustrate different kinds of potential hazards related to synthetic biology (Figure 1). If the life is truly artificial - for example, if it is built from one of the weird genetic alphabets where the DNA has six different kinds of nucleotides (G, A, C, T, Z and P) that we have developed, then it is less hazardous to us as a pathogen; human beings would not be particularly nutritious, from its viewpoint. Clearly, Venter's bug has all three of the attributes illustrated in Figure 1 that are potentially dangerous: it is built of the same stuff as we are, it is self-sustaining, and it can evolve. But since it is essentially identical to a bacterium that already exists, the danger it presents is hardly new. Further, bugs that have been partly synthesized by humans, throughout the past 30 years of synthetic biology, have never been able to survive well in competition with bugs that have had the advantage of billions and billions of years of Darwinian evolution.

## *The Economist *claims that with Venter's synthesis of a cell, a 'new era of synthetic biology is dawning'. Is this true?

Certainly not. Synthetic biology has been with us ever since recombinant DNA technology first allowed biologists to synthesize new forms of life - at least in the sense of constructing new DNA molecules and putting them into cells. In fact, the term 'synthetic biology' was coined in 1974 by Waclaw Syzbalsky to describe the application of recombinant DNA technology to generate organisms with new genetic properties. What Venter demonstrated is that it can be extended to replacement of the entire genome of a cell, not just changing parts of it.

## Is there value in doing this synthesis?

Yes, in many ways, but perhaps most important, synthesis can complement observation, controlled perturbation, and analysis in a science. Technology to enable synthesis has been available to chemists for more than a century, and has contributed to nearly every advance in chemical theory. For example, nearly all of our understanding of the chemical behavior of enzymes, metabolisms, and even diseases has come with the help of chemically synthesized molecules. Synthesis, in turn, allowed chemistry to complete its central paradigms faster than fields lacking synthesis. Fields lacking synthesis include astrophysics, cosmology, and planetary science. Imagine how much faster these fields might advance if we could synthesize new planets, stars or new universes to test their theories. The planet of Magrathea, whose inhabitants, according to *The Hitchhiker's Guide to the Galaxy*, accumulated fabulous wealth building planets to order, sadly does not exist.

Biology has historically also lacked synthetic technology - at least until the 1970s, with the advent of recombinant DNA technology. At first, biologists used biotechnology to cut and paste single genes, rearranging what was found naturally to modify living systems. In the 1980s, however, synthetic biologists moved away from nature, synthesizing entire genes encoding proteins, generating new artificial genetic systems with extra nucleotide letters, and engineering the expression of proteins with more than 20 different kinds of amino acids. These have already had an impact - for example, 'GACTZP' DNA (DNA built from the natural G, A, C, T nucleotides as well as our synthetic Z and P nucleotides) is, in one of its forms, incorporated into diagnostics assays that measure the load of HIV virions in patients at risk of AIDS. Here, the fact that extra 'letters' in the DNA alphabet do not bind to natural nucleotides allows the clinical assay to detect viral DNA without interference from natural DNA. Today, clinical diagnostics tools based on our synthetic genetic systems help personalize the care of some 400,000 patients annually worldwide. Curious readers will find a 2004 review I wrote on some of these applications listed below.

## Where can I find out more?

### Books

Adams D: *The Hitchhiker's Guide to the Galaxy*. London: Pan Books; 1979.

Benner SA: *The Life, the Universe and the Scientific Method*. Gainesville: FfAME Press; 2009.

### Articles

Anonymous: **Genesis redux**. *The Economist *2010, **395**:81-83.

Benner SA: **Understanding nucleic acids using synthetic chemistry**. *Accounts Chem Res *2004, **37**:784-797.

Benner SA, Hutter D: **Phosphates, DNA, and the search for nonterrean life. A second generation model for genetic molecules**. *Bioorg Chem *2002, **30**:62-80.

Cleland CE, Chyba CF: **Defining 'life'**. *Origins Life Evol *2002, **B32**:387-393.

Edge MD, Greene AR, Heathcliffe GR, Meacock PA, Schuch W, Scanlon DB, Atkinson TC, Newton CR, Markham AF: **Total synthesis of a human-leukocyte interferon gene**. *Nature *1981, **292**:756-762.

Gibson DG, Glass JI, Lartigue C, Noskov VN, Chuang RY, Algire MA, Benders GA, Montague MG, Ma L, Moodie MM, Merryman C, Vashee S, Krishnakumar R, Assad-Garcia N, Andrews-Pfannkoch C, Denisova EA, Young L, Qi ZQ, Segall-Shapiro TH, Calvey CH, Parmar PP, Hutchison CA 3rd, Smith HO, Venter JC: **Creation of a bacterial cell controlled by a chemically synthesized genome**. *Science *2010 [Epub ahead of print].

Joyce GF: *Origins of Life: The Central Concepts*. Edited by Deamer DW, Fleischaker GR. Boston; Jones and Bartlett; 1994.

Nambiar KP, Stackhouse J, Stauffer DM, Kennedy WG, Eldredge JK, Benner SA: **Total synthesis and cloning of a gene coding for the ribonuclease S protein**. *Science *1984, **223**:1299-1301.

Switzer CY, Moroney SE, Benner SA: **Enzymatic incorporation of a new base pair into DNA and RNA**. *J Am Chem Soc *1989, **111**:8322-8323.

Szybalski W: ***In vivo *and *in vitro *initiation of transcription**. In *Control of Gene Expression*. Edited by Kohn A, Shatkay A. New York: Plenum;1974: 23-24, 404-405, 411-412, 415-417.

Wang L, Brock A, Herberich B, Schultz PG: **Expanding the genetic code of *Escherichia coli***. *Science *2001, **292**:498-500.

Yang Z, Chen F, Chamberlin SG, Benner SA: **Expanded genetic alphabets in the polymerase chain reaction**. *Angew Chem *2010, **49**:177-180.

